# PLOS-LLM: Can and should AI enable a new paradigm of scientific knowledge sharing?

**DOI:** 10.1371/journal.pdig.0000501

**Published:** 2024-04-25

**Authors:** Robert C. Hughes, Alastair van Heerden

**Affiliations:** 1 Maternal and Child Health Intervention Research Group, London School of Hygiene and Tropical Medicine, London, United Kingdom; 2 Centre for Community Based Research, Human Sciences Research Council, Pietermaritzburg, South Africa; 3 SAMRC/Wits Developmental Pathways for Health Research Unit, University of the Witwatersrand, Johannesburg, South Africa; Hospital Clínic de Barcelona: Hospital Clinic de Barcelona, SPAIN

Most people agree that the traditional academic publishing model is not working [1,2]. Even editors of prestigious journals have been saying this for some time. There is, however, less consensus about which of the many problems are most pressing. Is it paywalls limiting access, especially for those outside of rich countries/institutions? Or is the inefficiency inherent in re-formatting papers as they bounce between dated online submission systems with login systems from the last century? Perhaps the key problem is the way that journal editors, with variable urgency, decide what to publish in their prestigious journals, juggling commercial pressures to attract attention/clicks/advertising with attempts to identify what research is most important/transformational? Add in that the market is dominated by a tiny number of publishers–in 2013 for example, over half of all published papers were published by just five publishers [3]–and it is perhaps unsurprising that it’s getting harder and harder to attract peer reviewers [4], further slowing the system down. And this is without even getting into the predatory business models of the less scrupulous journals [5]. What is perhaps even more concerning is how these problems are likely to influence not just the dissemination of research, but the whole system of scientific inquiry from decisions about what ‘publishable’ work to focus on to which results to and do not get into the public domain.

The ongoing shift towards open access publication represents a set of important, yet far from inclusive [6], changes in the academic publishing landscape as do the rise in use of pre-prints, emerging models of transparent, progressive, ‘post-publication’ peer-review. Social media is also radically changing how, and what, science is shared. But is it time for a much more radical disruption to the ecology of scientific knowledge? In this article, having touched on some of the many current problems with academic publishing, we propose a new model for scientific knowledge sharing. We believe that emerging technologies, specifically foundational large-language models [7] trained with human supervision and supported by semantic search, may enable a radical re-thinking of what the ‘journal of the future’ might be. We hope to provoke discussion and debate about how such a revolution could, and should, be more open, transparent and efficient; something that–unlike the status quo–is fit for the 21^st^ century, benefiting both researchers and all of society.

It is worth beginning by remembering that–although journals do sometimes feel like they have been around forever–there was a time when a different model dominated. Scientific journals as we known them were introduced in 1665 with the publication of the journals including the Journal des Sçavans in France and the Philosophical Transactions of the Royal Society of London in England. They were founded to advance scientific knowledge by building on colleagues’ results, aiming to avoid duplication in an endeavour to establish scientific priority setting. Prior to this introduction of journals, scholarly communication occurred through personal correspondence, learned society meetings, and books. The advent of journals enabled a more structured and regular distribution of scientific knowledge, which facilitated systematic recording and archiving of scientific advancements [3]. What is perhaps surprising is just how similar a model remains in place today.

Since the release of ChatGPT in late 2022 awareness about large language models [LLMs] has gone from niche interest to widespread use, intrigue and also concern [8]. While some view LLMs as a positive, transformative technology that could free authors from mundane writing tasks and allows them to focus on more complex aspects of their work, others are calling for a ban on its use and dismissing it as low-quality plagiarism [9]. There are valid concerns about the quality and reliability of AI-generated content, as exemplified by the case of Galactica, a short-lived experimental project by Meta designed to assist scientists with relevant scientific compositions. Interestingly, after 3 days it was taken down due to concerns about the model confidently spouting unsubstantiated facts [10]; a common problem for many current LLMs, namely their propensity to “hallucinate”–often very plausibly [11]. Despite these concerns, there is a consensus among commentators that ChatGPT represented a significant advancement in natural language processing and had the potential to revolutionize the field of AI writing. It is notable that 80% of scientists recently polled said they had used AI chatbots such as chatGPT [12]. While most debate focuses on the role of LLMs in scientific writing [13] we propose that next-generation LLMs could–with human supervision–be trained to radically disrupt the whole system of scientific inquiry and knowledge sharing. In replacing journals, could they make papers and systematic reviews look as outdated as telegrams and fax machines?

Here is how we think it might be able to work. Instead of submitting your carefully worded paper that you’ve taken months, or even years, to craft, as soon as the study receives ethical approval the methods and results are—with context [setting, detailed protocols, related studies], but without any ‘fluff’—submitted for inclusion into the model; let’s call it “PLOS-LLM”. This would replace publication of a protocol paper and, where relevant, registration of a trial. Later, the results, and dataset, are submitted too. Emerging multi-modal models mean that there are few limits to the types of data that could be submitted to the model; all of raw observations, tables, figures and even images and audio could be shared [14]. Initially human supervision, including historic human supervision in the form of the peer-reviewed literature, would be critical. Humans could act as a ‘gatekeepers’ to new science being ‘fed into’ the model. But the LLM would help with this too—finding relevant recent similar research, identifying the most relevant experts to evaluate the appropriateness of methods for example, and allowing them to, using natural language, query, rather than read the submission. Humans might still then need to decide on how these results should be included, perhaps scoring them—openly—on rigor/creativity/novelty, allowing these scores to inform how the research is subsequently presented, and what caveats are applied.

However, even much of this review process would gradually be increasingly automated. The LLM will, as it grows, be able to spot many of the errors that are so often missed by peer reviewers, and in seconds not weeks/months/years. But humans will—for the foreseeable future at least—need to be in the loop too; we think this machine-human collaboration will remain vital.

The advantages of such a model are as broad as they are radical. Combined with emerging semantic search technologies (a mathematical approach to search that uses the meaning of what you’re looking for to find related things, even if you don’t use the exact words to describe it) could mean that the whole body of literature on a topic can almost effortlessly be queried in real time with scientific advances. Rather than database searching and then either relying only on an abstract and/or navigating numerous paywalls, anyone could query the model for the specific questions they’re looking for. The time spent on laborious, yet instantly out-of-date, systematic reviews could be directed elsewhere. As illustrated in Fig 1, when querying the model it would be possible to expand and contract the level of detail, ’zooming in’ from headline findings to raw research data from individual studies. Meta-analyses would always be up to date; re-running as soon as new data are inputted and checked. There would be no more re-formatting of papers, uploading tracked changes, sending (over)polite letters setting out the detail of edits. All model inputting and training could be through a simple chat-based interface (perhaps using voice to text to free us from our keyboards/screens?). The model may also be able to identify promising research avenues that humans have missed, at the same time suggesting methods to explore them.

**Fig 1:**
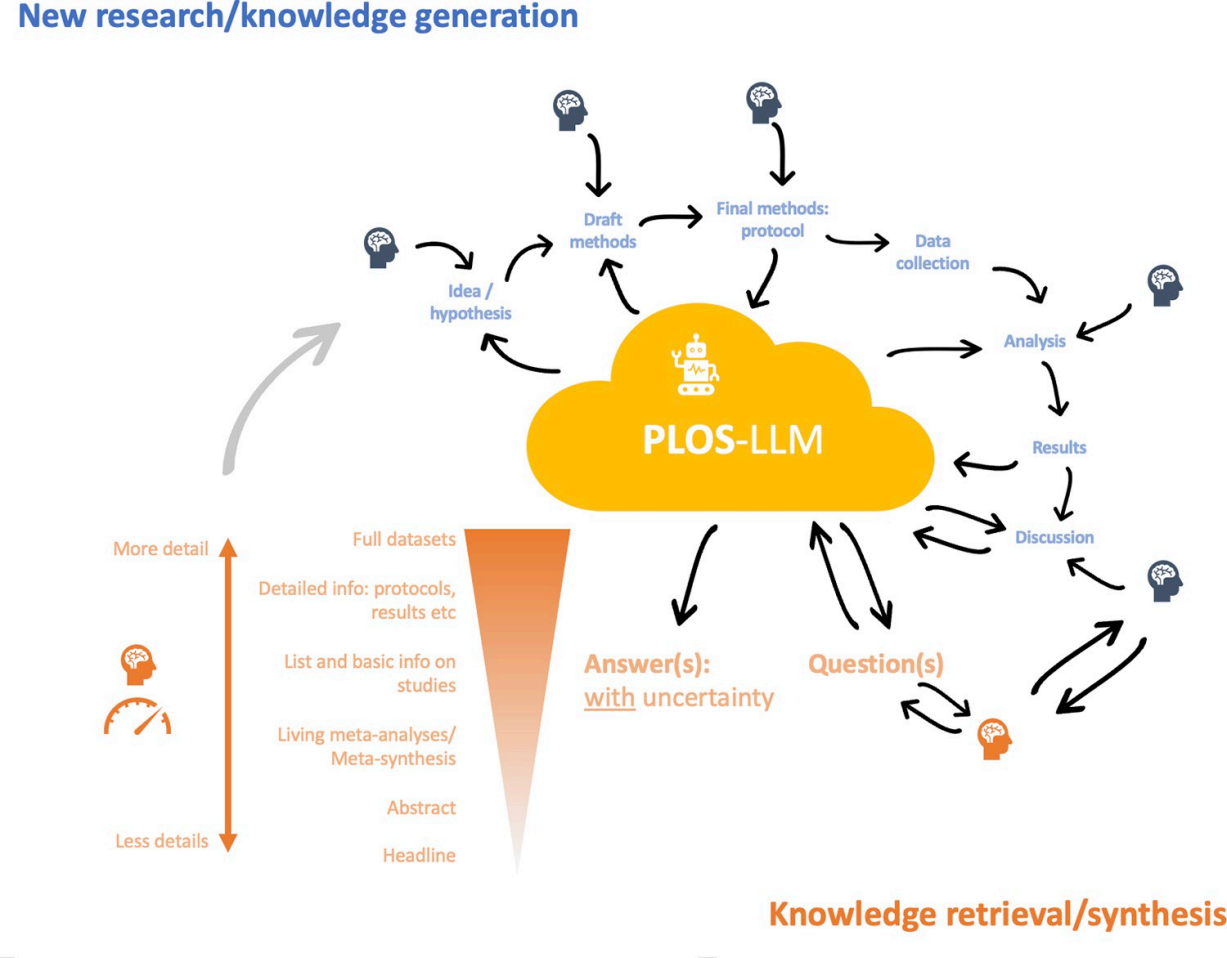
Schematic illustration of how “PLOS LLM” could interact with humans, considering both knowledge generation and retrieval/synthesis processes. Text in blue illustrates how new **research/knowledge generation** could interface with PLOS-LLM, and the steps in orange illustrate how **knowledge retrieval/synthesis** could work, for example allowing users to ‘zoom in and out’ from looking at headline answers to a question, all the way down to exploring the datasets of individual studies.

Tools such as elicit.org provide insights into how this vision may eventually look. Employing large language models, Elicit aims to help scientists automate various research workflows, such as parts of the literature review process. With Elicit, researchers can find relevant papers even if the keywords do not match perfectly and can receive summaries of the papers that are specific to their questions. Additionally, Elicit has the capability to extract key information from papers, further streamlining the research process. In addition, it is already easy to use GPTs turn any paper or website into a chatbot that you can ‘talk with’ rather than reading in full: https://shorturl.at/cglJQ

However, such a vision for the future of scientific knowledge generation and sharing does bring potential risks. The finetuning of model weights will be critical; even more critical than the biases of a journal editor, omission, misunderstanding, and errors in how the model works could have far-reaching, and sometimes hidden, consequences. For example, if the model is developed with or develops biases or blind-spots this could lead to systematic exclusion of some science or scientists. Alignment research would be critical to ensure the system’s goals, behaviours, and knowledge were in accordance with the world view(s) of the scientific community, perhaps requiring new efforts to build consensus on ‘the point’ of scientific research. In addition, questions about who develops and controls such a model, or set of models, will be as vital as–or perhaps even more important than–the current debates around who owns academic publishing.

Furthermore, most of us can–warts and all–understand the model of a peer-reviewed journal. The prospect of a powerful LLM that we cannot ‘look under the bonnet’ of is undeniably unsettling. How could and should trust in such models be built, and how should they be evaluated? And finally, who pays? Is there, and should there be, a business model here, or could development of this sort of global public good fit the bill for the sort of thing that governments (and or seemingly ever-richer philanthropists) could be persuaded to finance?

The disruption coming with emerging AI technologies ought to be an opportunity to design out some of the biases, inequalities and inefficiencies of the status quo of scientific knowledge sharing, but doing so will require a concerted effort alongside an acknowledgment of the new risks that may emerge. For example, today, much AI innovation is centred on the Global North, especially Silicon Valley in the USA and, to a lesser extent, in China. Incorporating AI into our models for scientific knowledge generation and sharing will present a set of tricky questions about control and transparency, all of which are as important as they are complicated. With the pace of development of these sorts of tools accelerating, we feel that a deeper conversation about them within and beyond academia is overdue, and hope that the potential version of the future that we have presented here can help to stimulate these debates. That said, given the state of our current model, there is plenty of room for improvement.

## Declaration of generative AI and AI-assisted technologies in the writing process

During the preparation of this work the authors used ChatGPT4.0 in order to review the readability of the draft and to provide some critique which was then considered by the authors. After using this tool/service, the authors reviewed and edited the content as needed and takes full responsibility for the content of the publication.
